# Exceptionally Stable CH_3_NH_3_PbI_3_ Films in Moderate Humid Environmental Condition

**DOI:** 10.1002/advs.201500262

**Published:** 2015-09-25

**Authors:** Baohua Wang, Tao Chen

**Affiliations:** ^1^ Department of PhysicsThe Chinese University of Hong Kong Shatin, N.T.Hong KongChina

**Keywords:** chemical vapor transport, moisture stability, perovskite, photovoltaic devices, solar cells

## Abstract

**An unprecedentedly stable CH_3_NH_3_PbI_3_ film synthesized by a modified chemical vapor transport method** is demonstrated. The results show that the crystal structure, light absorption, and device efficiency do not degrade after storing for 100 d in air with 40% relative humidity, while the conventional solution‐processed perovskites are usually stable for less than 20 d in similar conditions.

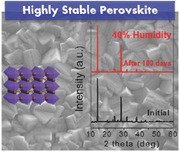

Methylammonium lead iodide (CH_3_NH_3_PbI_3_) based perovskite materials have drawn intense interests due to the excellent photovoltaic energy conversion capability; the power conversion efficiency (PCE) of perovskite solar cell has been boosted to 20.1%.^1^ Recent investigations have gained comprehensive understandings about the materials and operational principles as well as accumulated rich experiences in the device fabrication.^2^, ^3^ It has been increasingly acknowledged that the major concern regarding this technology is the poor device stability.^4^ Most recently, an extensive investigation shows that in 25% relative humidity (RH) the absorbance of the perovskite CH_3_NH_3_PbI_3_ film decays to half of its original value in 57 d (*t*_1/2_). While in moderate moisture (40% RH), the *t*_1/2_ is only 26 d. The high sensitivity to moisture poses severe challenge in terms of the practical applications.^5^

To improve the stability, one way is to protect the perovskite from water molecule attachment. In this regard, Niu et al. utilized Al_2_O_3_ as an interfacial coating layer to resist the moisture permeation; the stability can be improved by a few days.^6^ Han's group employed a thick layer of carbon materials as both hole transporting materials (HTMs) and protection layer to improve the moisture stability; the final PCE reached 12.3% and maintained for over 40 d.^7^ Inorganic HTMs such as CuI and NiO*_x_* could also enhance the stability by a few days.^8^ The other approach is to improve intrinsic stability of the perovskite. Apparently, this solution is of fundamental significance and can alleviate the reliance on the stringent encapsulation, thus reducing the fabrication and installing expenses of solar panels. In this perspective, Seok's group found that doping Br in the iodide‐perovskite to form CH_3_NH_3_PbI_3‐*x*_Br*_x_* could noticeably improve the stability to 20 d.^9^ Most recently, a layered perovskite (C_6_H_5_(CH_2_)_2_NH_3_)_2_(CH_3_NH_3_)_2_[Pb_3_I_10_] was synthesized which was stable for 45 d in ambient condition; the device delivered a PCE of 4.7%.^10^

Here we demonstrate that the intrinsic stability of CH_3_NH_3_PbI_3_ film can be dramatically improved by tailoring the compositional purity and morphology of the perovskite film through a modified chemical vapor transport (mCVT) reaction approach (**Figure** **1**a). In this process, PbI_2_ film is firstly prepared as condensed phase by spin‐coating PbI_2_ solution (in *N*,*N*‐dimethylformamide, DMF) onto the TiO_2_‐compact‐layer‐coated fluorine‐doped tin oxide (FTO) and dried at 100 °C for 10 min. Scanning electron microscope (SEM) characterization shows that the film is composed of small crystallites hosting many randomly distributed pores (Figure 1b and Figure S1, Supporting Information). Afterward, the PbI_2_ film is transferred to the tube furnace containing CH_3_NH_3_I powder. The reaction between PbI_2_ film and CH_3_NH_3_I is conducted at 140 °C at a pressure of 1 mbar using Ar as carrier gas (Figure 1a). Optimizations show that appropriate reaction time is 2–3 h. Reaction less than 2 h cannot lead to complete transformation to CH_3_NH_3_PbI_3_ while elongated reaction brings forth poorer device efficiency (Figure S2, Supporting Information).

**Figure 1:**
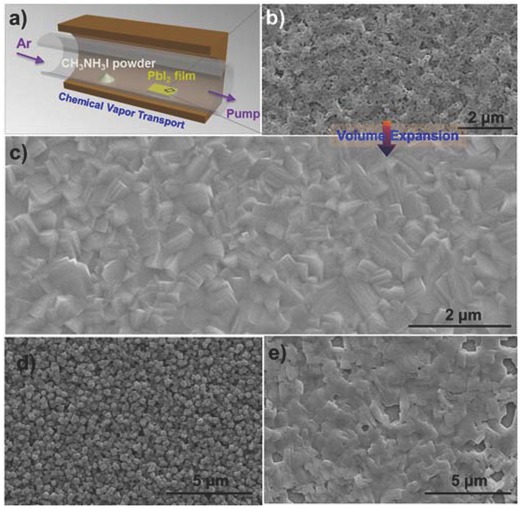
a) Experimental setup of the chemical vapor transport reaction, where Ar is used as carrier gas and the reaction is conducted at 140 °C in an isothermal furnace. b,c) SEM images of the as‐synthesized PbI_2_ porous film and perovskite film synthesized via the reaction between the PbI_2_ film and CH_3_NH_3_I vapor using the experimental setup in (a). d,e) SEM images of the perovskite film synthesized by the conventional two‐step deposition method and one‐step solution process, respectively. Large‐area SEM images are provided in the Supporting Information.

SEM image (Figure 1c) of the as‐prepared film exhibits tightly packed crystals without pinholes throughout the whole film (denoted as sample 1). A large‐area SEM image is provided in Figure S3 (Supporting Information), showing pinhole and crack free across the surface. Obviously, the volume expansion upon the formation CH_3_NH_3_PbI_3_ is responsible for diminishing pores in the original PbI_2_ film. Previously, the solid–gas reactions in either static gas atmosphere or two‐zone furnace generate pinholes or cracks in between the perovskite nanocrystals.^11^ Here the mCVT reaction in isothermal chamber with carrier gas is thus advantageous. The other distinct feature of the mCVT approach is that the flowing gas could more efficiently deplete excess CH_3_NH_3_I deposition on the surface of the as‐prepared film than the static gas atmosphere or the two‐zone apparatus.

The phase purity of the as‐prepared film is characterized by X‐ray diffraction (XRD), displaying typical perovskite structure of CH_3_NH_3_PbI_3_ without impurity peaks (**Figure** **2**a). To gain clear and reliable conclusions regarding the moisture stability of perovskite, we record the phase changes in 40% RH in air under darkness in order to rule out other potential influences such as UV light. As a result, the XRD characterizations show identical patterns after storing for 30, 45, and 100 d (Figure 2a). The UV–visible absorption characterizations also show nearly the same spectra after storage (Figure 2d). It should be noted that the use of PbCl_2_ and CH_3_NH_3_I as precursors in mCVT system can generate identical film morphologies (Figure S4, Supporting Information) with the same stability to that synthesized with PbI_2_ and CH_3_NH_3_I as precursors.

**Figure 2:**
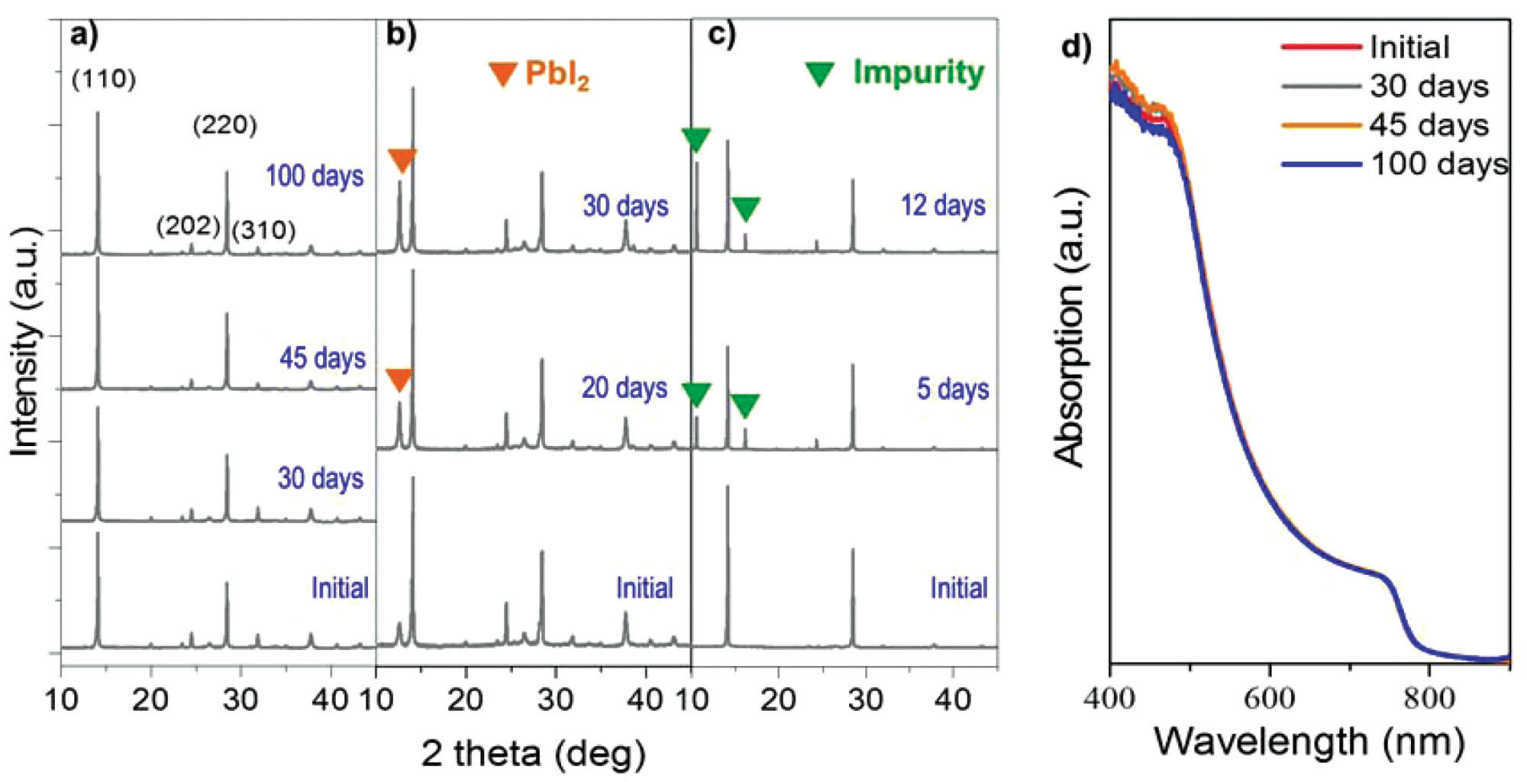
a) XRD characterization of the perovskite films prepared by mCVT (sample 1), b) two‐step solution processing using PbI_2_ and CH_3_NH_3_I (sample 2), and c) one‐step processing using PbCl_2_ and CH_3_NH_3_I as reactants (sample 3). The samples are stored in air with 40% RH under darkness. d) UV–vis absorption spectra of the mCVT prepared CH_3_NH_3_PbI_3_ storing in air with 40% RH for up to 100 d.

To uncover the reason why the mCVT‐synthesized perovskite exhibits unusual stability, we prepare perovskite by a solution process with the same precursors for comparative analysis. In brief, PbI_2_ is first spin‐coated on the TiO_2_/FTO substrate; it is then dipped into the CH_3_NH_3_I solution. Afterward, the film is taken out and heated at 100 °C for 10 min. This is the conventionally applied “two‐step sequential deposition” method (denoted as sample 2).^2^ The final film is composed of cuboid nanocrystals (Figure 1d). XRD characterization shows typical pattern of CH_3_NH_3_PbI_3_ (Figure 2b). The moisture stability of the film is examined by storing with 40% RH at the same condition as sample 1. According to the XRD analysis, the diffraction peaks from PbI_2_ increase substantially after 20 and 30 d, indicating that the perovskite starts to decompose in less than 20 d. The degradation speed is in agreement with the literature reports.^5^, ^10^ We also monitor the degradation process of the perovskite film under irridiation and compared it with that of the mCVT‐prepared one. It is found that the mCVT‐prepared film is still much more stable than the two‐step prepared one except that the decomposition rate of both the films is more quickly under irridiation than in darkness.

The difference in the synthesis of sample 1 and sample 2 is that the reaction for sample 2 is in DMF solution followed by annealing for 10 min at 100 °C. A fact is that DMF can coordinate with Pb^2+^ and it is verified that the DMF molecule is prone to intercalating between the perovskite nanocrystals or adsorbed onto the surface of solution processed perovskite film.^12^, ^13^ The intercalation of DMF molecules could create microgaps onto the perovskite nanocrystals, generating more available sites for water molecules attachment (**Scheme** **1**a). Therefore, the decomposition by means of hydrolysis can be considerably expedited. To substantiate this assumption, we prepare CH_3_NH_3_PbI_3_ film by the two‐step method and intentionally dry it at lower temperature (70 °C) with reduced annealing time. Trace amount of DMF is detected in the perovskite film with Fourier transform infrared spectroscopy at this condition (Figure S5, Supporting Information). Lower temperature annealing could lead to more DMF retaining in the film and thus more available site for water attachment. The as‐prepared perovskite shows identical crystal structure to that annealed at 100 °C for 10 min, while the decomposition rate is much faster than that annealed at high temperatures (Figure S5, Supporting Information), it decomposes nearly completely after 30 d. On the other hand, if we prolong the annealing time to detach the DMF molecules, the stability can be improved to certain extent depending on the annealing time.

**Scheme 1:**
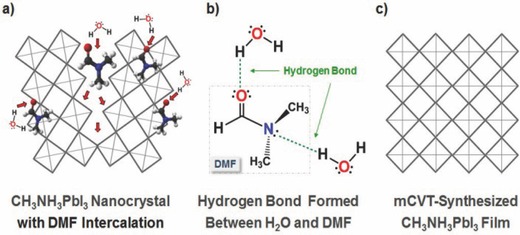
a) Illustration of DMF molecules intercalated into or on the surface of CH_3_NH_3_PbI_3_ nanocrystal which is synthesized via conventional solution process using DMF as the solvent. The red arrows indicate possible sites for water attachment. b) The formation of the hydrogen bond between water molecule and DMF. c) DMF‐free CH_3_NH_3_PbI_3_ nanocrystals synthesized by the mCVT approach. The CH_3_NH_3_^+^ is omitted in (a) and (c) for clarification.

This is an open access article under the terms of the Creative Commons Attribution License, which permits use, distribution and reproduction in any medium, provided the original work is properly cited.

Furthermore, since DMF molecule possesses both O and N, these two atoms can form hydrogen bonding with H_2_O molecule (Scheme 1b), which promotes H_2_O attachment. Hence, the existence of the DMF in the perovskite brings forth two negative effects: (1) intercalating between the perovskite crystals to generate more available areas of the perovskite films for H_2_O attachment and (2) accumulating H_2_O molecule through hydrogen bonding, on both the surface of perovskite film and gaps generated (Scheme 1a).

On the contrary, in the mCVT method, the 2 h reaction in high‐vacuum condition at 140 °C is able to detach the DMF much more efficiently from the film; the formation of this kind of structural flaw can be efficiently suppressed (Scheme 1c). In addition, the overall exposed area of sample 1 is much smaller than that of sample 2 (Figure 1c,d), this is also a significant factor slowing down the hydrolysis process. The phenomenon that the compact morphology of the perovskite film is beneficial for the stability enhanced is also reported.^14^ Here, we find that the phase purity of the perovskite film also prominently affects the film's stability.

Conventionally, the planar perovskite film is prepared by a casting‐and‐annealing processing approach; here we also prepare the film for a comprehensive study. A mixture solution of PbCl_2_ and CH_3_NH_3_I in DMF is usually used as the reaction precursors, which is spin‐coated onto the TiO_2_ compact layer. It is then heated at 100 °C for 45 min. SEM image (Figure 1e) shows relatively uniform film with a considerable number of pinholes (sample 3), which is in agreement with the literature report.^15^ Obviously, the existing pinholes can serve as channels for water attachment. On the other hand, it is proved that the reaction between PbCl_2_ and CH_3_NH_3_I releases gas in the forms of CH_3_NH_3_Cl, CH_3_NH_2_, or HCl,^13^, ^16^ which can induce micropores in the crystals. Therefore, the surface area for water adsorption is substantially increased. It is also plausible that there are DMF molecules on the film surface and unreacted chlorine remained in sample 2 (Figure S9, Supporting Information) that aid the water attachment. Therefore, sample 3 degrades very quickly (Figure 2c) and changes to translucent after only 5 d in air with 40% RH. The appearance of diffraction at 10.54° indicates the formation of (CH_3_NH_3_)_4_PbI_6_·2H_2_O as a result of water adsorption.^5^

The device (with samples 1, 2, and 3 as the light absorbing layers) performance was examined on the ground of planar heterojunction architectures as shown in **Figure** **3**a,b, where spiro‐OMeTAD is utilized as HTM and thermally evaporated Ag is employed as metal contact. A cross‐section of the device based on mCVT‐synthesized CH_3_NH_3_PbI_3_ is shown in Figure 3b. The initial PCE of sample 1 is 12.23%. The devices based on samples 2 and 3 generate PCE of 12.11% and 12.74%, respectively (Figure 3c). It is observed that the *V*_oc_ of device based on mCVT fabricated film is 0.95 V, which is slightly lower than the two‐step and one‐step solution processed ones which are 0.97 and 1.00 V, respectively. It is possible that defect states are generated at elevated temperature in an I‐rich environment in the mCVT synthesis, which usually leads to slight voltage loss.^17^

**Figure 3:**
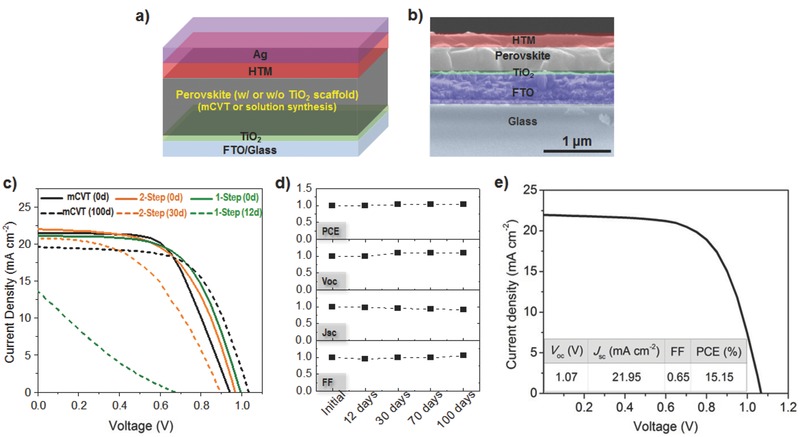
a) Device configuration of the perovskite solar cells, in which the perovskite films were prepared by either the mCVT method or the conventional solution processing. b) A cross‐section image of the solar cell based on mCVT synthesized perovskite film. c)Photocurrent–voltage characteristics of the device based on perovskite film fabricated by the mCVT, two‐step deposition method, and one‐step deposition method; the solid and dashed lines indicate initial device performance with respect to the film stored for 100, 30, and 12 d, respectively. d) The evolutions of normalized PCE, *V*_oc_, *J*_sc_, and FF of the devices based on mCVT prepared films. e) *J–V* curve of the best performance solar cell based on mCVT‐fabricated CH_3_NH_3_PbI_3_ film after storing in air with 40% RH for 30 d.

To exclude the influence of the other components such as the HTM on the device stability and gain a clear conclusion regarding the stability of perovskite film, we test the photovoltaic performance of the aged perovskite films by using fresh HTM every time. The performance evolution of the mCVT‐synthesized perovskite is shown in Figure 3c,d. After storing the perovskite film for 12 d in ambient condition, the performance has no obvious change, showing PCE of 12.25%. The PCE (12.68%) is even higher after storing the film for up to 30 d with the *V*_oc_ increasing substantially from 0.95 to 1.05 V, the *J*_sc_ slightly drops 5%, and there is no much variation in fill factor (FF) on average. These alternations finally render an increment on the overall PCE. Remarkably, after storing for 70 and 100 d, the devices show PCEs of 12.59% and 12.71%, respectively. The *J–V* curves are provided in Figure S6 (Supporting Information).

Notably, the device efficiency is increased in the first 30 d and the highest PCE of 15.15% is obtained (Figure 3e). There are dual mechanisms for the efficiency improvement. First, the perovskite film is prone to decomposing to PbI_2_ even though with negligible concentration. The formation of PbI_2_ is favorable for a larger *V*_oc_, which has been confirmed by fabricating perovskite with residual unreacted PbI_2_ (Figure S2, Supporting Information). The type‐I heterojunction between PbI_2_ and perovskite is the reason for the enlarged *V*_oc_.^18^ Another mechanism contributing to the PCE improvement might originate from the defect state diminishing. As discussed before, the defect states form at high temperature in an iodine‐rich environment and they might self‐heal after storing for a long period.^17^

The photovoltaic performance of the device based on solution processed perovskite degrades quite fast. After storing for 12 d, the PCE of the one‐step perovskite drops to less than 20% of its initial value (Figure 3c). The *V*_oc_ and FF show significant reduction. In the two‐step‐prepared film, the device efficiency degrades to less than 75% of the initial value after 30 d (Figure 3c), which is more stable than the one‐step‐solution‐processed film while still much worse than the mCVT‐prepared film.

In conclusion, for the first time we show that perovskite film is stable for 100 d in air with 40% RH. The synthesis by an mCVT reaction in an isothermal furnace is able to generate high‐quality perovskite film when compared with the two‐zone furnace or the solid–gas reaction in static atmosphere. The conventional solution processed perovskite film reduces its absorption intensity to a half of its initial value even in 0% RH after 76 d.^9^ Thus, our research is a quantum leap in the stability improvement of organic–inorganic perovskite‐based solar cells. We discover that the phase purity is important for the film stability; the film morphology and arrangement of the perovskite crystallites synergistically contribute to the enhanced stability. The mCVT approach is also adaptable. It has been initially confirmed by preparing lead‐free CH_3_NH_3_SnI_3_ via the reaction between SnI_2_ film and CH_3_NH_3_I vapor, showing tight arrangement of the CH_3_NH_3_SnI_3_ nanocrystals without pinholes across the film (Figure S8, Supporting Information). In all, this research provides a new, low‐cost, and adaptable fabrication method for the perovskite film synthesis with excellent stability. The mechanistic understandings regarding the intrinsic stability of the perovskite film would benefit further improvement on the life time of the devices for practical applications.

## Experimental Section

The film (CH_3_NH_3_PbI_3_) synthesis is conducted by a chemical vapor transport synthesis in an isothermal furnace (Figure 1a). Excess amount of CH_3_NH_3_I powder is placed at the upstream in the tube furnace together with the PbI_2_‐covered substrate. The reaction is performed at 140 °C and 1 mbar with Ar as carrier gas. The details are included in the Supporting Information. It is worth noting that the reaction taking place in low pressure and isothermal environment is critical for obtaining high‐purity perovskite film.

## Supporting information

As a service to our authors and readers, this journal provides supporting information supplied by the authors. Such materials are peer reviewed and may be re‐organized for online delivery, but are not copy‐edited or typeset. Technical support issues arising from supporting information (other than missing files) should be addressed to the authors.

Supplementary
