# Supplementary material for: Exceptionally Stable CH3NH3PbI3 Films in Moderate Humid Environmental Condition
Source: Adv Sci (Weinh). 2015 Sep 25;3(2):1500262. doi: 10.1002/advs.201500262 (PMC5054937; doi:10.1002/advs.201500262)
Supplement: Supplementary file 1 — Supplementary [file ADVS-3-0i-s001.pdf]

Copyright WILEY-VCH Verlag GmbH & Co. KGaA, 69469 Weinheim, Germany, 2015.

ADVANCED SCIENCE
Open Access

Supporting Information

for *Adv. Sci.*, DOI: 10.1002/advs. **201500262**

Exceptionally Stable CH_3 NH_3 PbI_3 Films in Moderate Humid Environmental Condition

*Baohua Wang and Tao Chen**

Copyright WILEY-VCH Verlag GmbH & Co. KGaA, 69469 Weinheim, Germany, 2013.

Supporting Information

Exceptionally Stable $CH_3NH_3PbI_3$ Films in Moderate Humid Environmental Condition

*Baohua Wang and Tao Chen**

1. Materials
2. Experimental Details
3. Characterization
4. Supporting Discussion
5. Supporting Figures
6. Supporting References

1. Materials

All chemical materials are purchased from commercial suppliers and used as received unless stated otherwise. CH_3NH_3I is synthesized according to a literature method[1] and dried in a vacuum oven at 60 °C for 10 h before using.

2. Experimental Details

$CH_3NH_3PbI_3$ synthesized by mCVT. The procedures for preparing the perovskite film with a modified chemical vapor transport reaction (mCVT) are as follows. Firstly, an FTO substrate (14 Ω/sq) is cleaned with ultrasonication in deionized water, isopropanol (IPA) and acetone in sequence. After drying with air flow, the substrate is further treated with oxygen plasma for 2 min. Then, a 50-nm-thick compact layer of TiO_2 is prepared on the clean substrate by spin coating acidic titanium isopropoxide (TIP, 97%) precursor solution (0.24 M TIP and 0.12 M HCl in ethanol) at 5000 r.p.m for 60 s, followed by annealing on a hotplate at 500 °C for 60 min. After cooling down to room temperature, the TiO_2/FTO substrate is covered with a layer of PbI_2 by spin coating PbI_2 solution (460 mg/mL 99% PbI_2 in anhydrous dimethyl formamide (DMF)) at 3000 r.p.m. for 60 s. The PbI_2 film is dried at 100 °C for 10 min before transferred into a tube furnace for the mCVT. Excess amount of CH_3NH_3I powder is placed at the upstream in the tube furnace together with the PbI_2-covered substrate. After the tube is pumped to a vacuum of 0.01 mbar, the furnace is gradually heated to 140 °C in 30 min and a flow of Ar at 100 s.c.c.m. starts to carry the CH_3NH_3I vapor downstream to react with PbI_2. During the reaction, the pressure in the tube furnace is maintained at 1 mbar. The reaction time is optimized as 2 h at 140 °C for just complete transformation from PbI_2 into $CH_3NH_3PbI_3$.

$CH_3NH_3SnI_3$ and $CH_3NH_3PbBr_3$ film synthesized by mCVT. The mCVT method is adaptable for synthesizing other perovskite materials such as $CH_3NH_3SnI_3$ (Fig. S8 and S12) or $CH_3NH_3PbBr_3$ (Fig. S13). For $CH_3NH_3SnI_3$ synthesis, 1M SnI_2 or $SnCl_2$ solution in DMF is spin coated on the TiO_2/FTO substrate at 3000 r.p.m. for 60 s. After dried at 100 °C for 10 min on a hotplate, the film is transferred into a tube furnace containing CH_3NH_3I powder. The optimized reaction condition is the same as described above which is at 140 °C for 2 h. $CH_3NH_3PbBr_3$ film can also be synthesized using $PbBr_2$ and CH_3NH_3Br as precursors. The procedures are similar except that the optimized condition in the tube furnace is at a temperature of 160 °C for 2 h.

Perovskite $CH_3NH_3PbI_3$ synthesis via solution approaches. Perovskite films are also prepared by the conventional two-step sequential deposition method[2] and one-step precursor deposition method[3] for comparing their stability. In the two-step approach, the PbI_2 film is firstly deposited on the TiO2/FTO substrate as described above and then the film is dipped into a methylammonium iodide solution (8 mg/mL in IPA) for 60 s. The color of the film turns from yellow to dark brown in several seconds upon contacting with the CH_3NH_3I solution. Then, the perovskite film is annealed at 100 °C for 10 min.

Alternatively, the perovskite film is also prepared with the one-step deposition method. A precursor solution containing 0.88 M $PbCl_2$ and 2.64 M CH_3NH_3I in DMF is spin-coated on the TiO_2/FTO substrate at 3000 r.p.m. for 60 s, followed by annealing at 100 °C for 45 min in a nitrogen-filled glovebox. Some one-step prepared perovskite films are further treated with the mCVT approach at 140 °C for 2 h to investigate the effect on stability.

Stability examination. In order to monitor the degradation process, the perovskite films are stored in the same ambient condition with a relative humidity (RH) of 40% in dark at room temperature and taken out for X-ray diffraction (XRD, Rigaku

SmartLab) characterization after certain days. The XRD patterns are taken at a rate of 10°/min with a step of 0.01° using a Cu K_α line operated at 40 kV 40 mA.

In order to gain further insight about the mCVT method and the stability of the perovskite film, we fabricate solar cells using the as-prepared or aged perovskite films. Specifically, a 200-nm-thick hole transport material (HTM) of doped spiro-OMeTAD is spin-coated on top of the perovskite film from a precursor solution (1 mL chlorobenze solution contains 72.5 mg spiro-OMeTAD, 42.7 μL 4-tert-Butylpyridine and 26.5 μL 520mg/mL lithium salt in acetonitrile) at 5000 r.p.m. for 60 s. The HTM layer is oxidized in air for 15 hrs before a 100-nm-thick silver is thermally evaporated on it. The active area of the solar cell is 0.12 cm^2 defined by the area of the silver anode.

3. Characterization

The morphologies of the perovskite films are characterized with Quanta 400 F Scanning Electron Microscope (SEM). The X-ray diffraction patterns of the perovskite films are recorded with Rigaku SmartLab X-ray Diffractometer equipped with CuK_α X-ray tube operated at 40 kV, 40 mA. The UV-vis absorption spectra are tested with Hitachi U-3501 UV/VIS/IR Spectrometer. The J-V characterization of the solar cells is conducted in a nitrogen-filled glovebox under the illumination of AM 1.5 G solar-simulated light with an intensity of 100 mW cm^{-1}. Two probes mounted on the micropositioners are put in touch with the FTO and the silver electrodes, respectively. A stepwise external voltage is applied across the two electrodes with a step of 0.05 V and a scan rate of 50 ms/step. An I-V hysteresis is observed in the planar structure solar cell yielding a typical discrepancy of ca. 2% in PCE with forward and reverse scan directions.

4. Supporting Discussion

It is also found that there is tiny amount (0.1%) of chlorine in the perovskite according to the right-shifted XRD peaks (details are provided in Fig. S9). If we further anneal the perovskite in CH_3NH_3I vapor, the replacing of Cl by I can induce volume expansion to shrink the pores and thus reduce the effective area for water attachment. Further annealing treatment is also able to eliminate the surface adsorbed DMF molecules. As expected, the moisture stability of the annealed film is significantly improved (Fig. S10).

By analyzing the sample preparation and characterizations, here we propose that the existence of Cl in the sample should be the driving force for the different decomposition pathways between one-step and two-step prepared perovskite films. We have estimated the concentration of Cl in the one-step-prepared perovskite to be about 0.1% based on the lattice constant decrease (Figure S9). In our experiment, we also observed that methylammonium chloride (MACl) or $MAPbCl_3$ is much more hygroscopic than the MAI or $MAPbI_3$ in ambient condition, so the one-step-prepared perovskite film with Cl residue is prone to facilitating water adsorption and the formation of hydrate such as $MA_4PbI_6{\cdot}2H_2O$. In contrast, the two-step-prepared perovskite film contains no Cl, it thus undergoes direct decomposition (into PbI_2) in absence of significant water attachment.

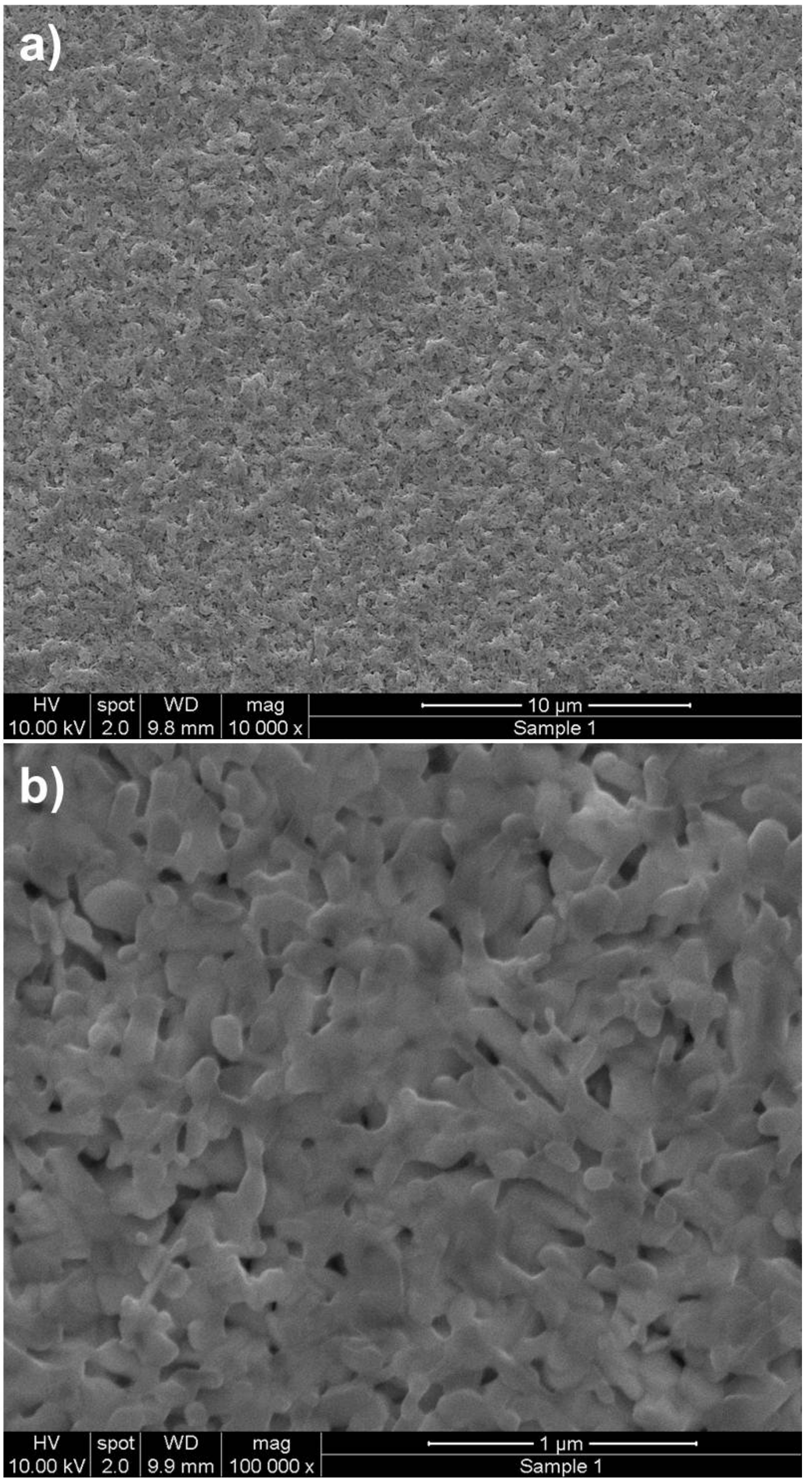

Figure S1 SEM images of the as-synthesized PbI_2 film with low (a) and high (b) magnifications.

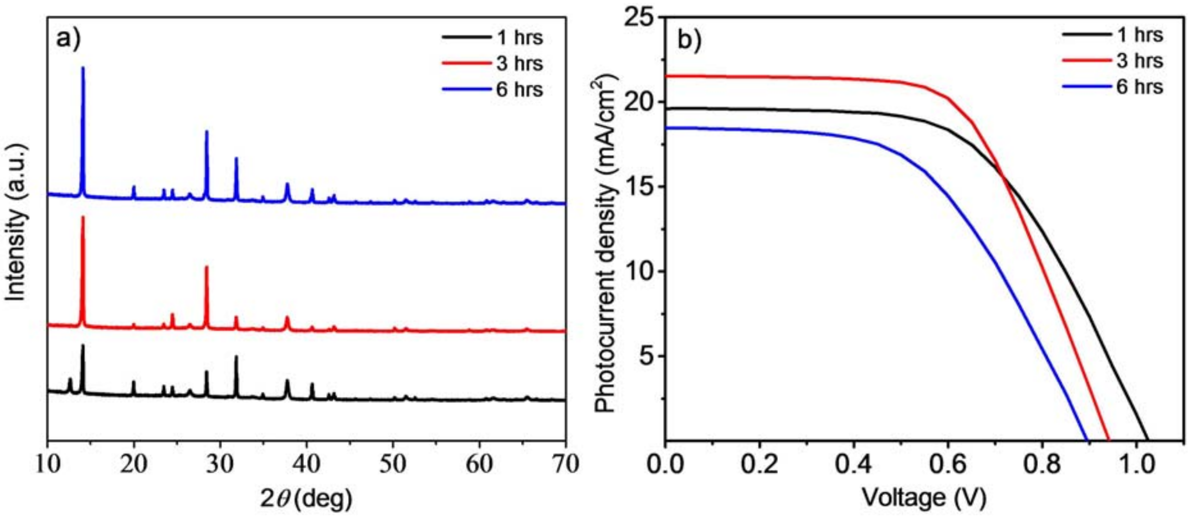

Figure S2 XRD patterns and J-V curves of mCVT-synthesized perovskite film with different reaction durations. a) XRD patterns of mCVT perovskite film with solid-gas reaction time of 1 h, 3 h and 6 h. b) J-V curve of the solar cells fabricated using mCVT perovskite films with solid-gas reaction time of 1 h, 3 h and 6 h.

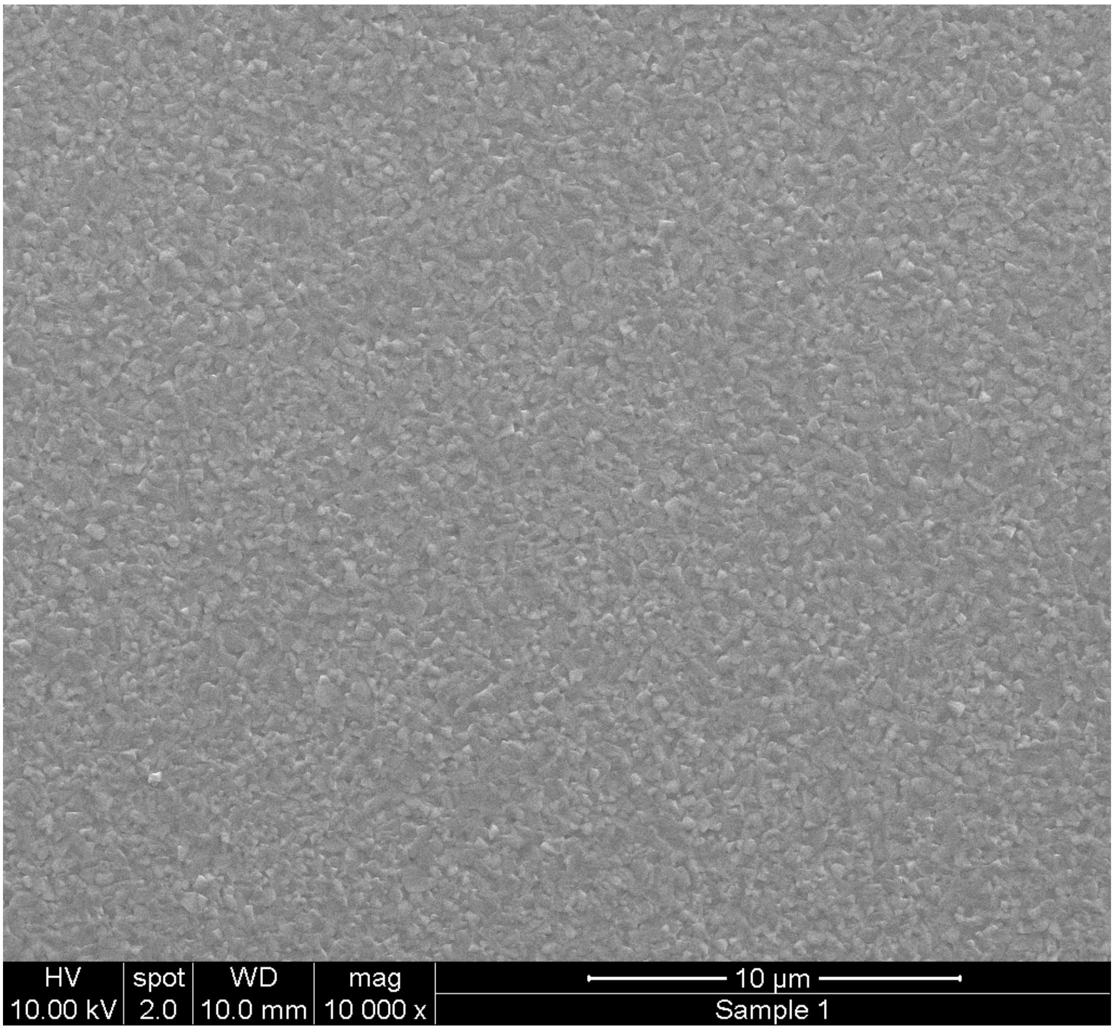

Figure S3 Large-area SEM characterization of the mCVT fabricated $CH_3NH_3PbI_3$ film, showing pinhole-free morphology.

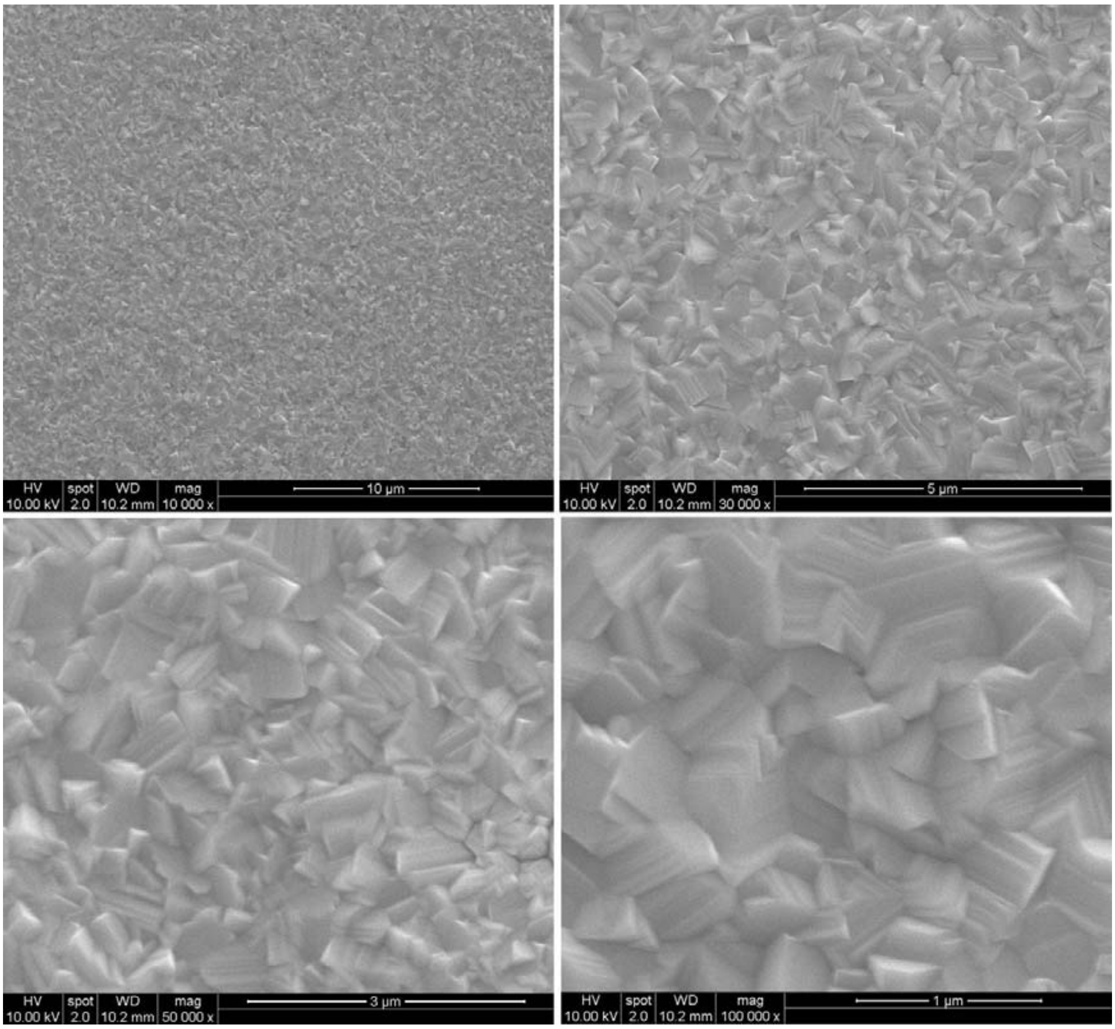

Figure S4 SEM images with different magnifications of the mCVT fabricated $CH_3NH_3PbI_3$ film by the reaction between $PbCl_2$ film and CH_3NH_3I vapor, showing identical morphology to that synthesized using PbI_2 film and CH_3NH_3I as reaction precursors.

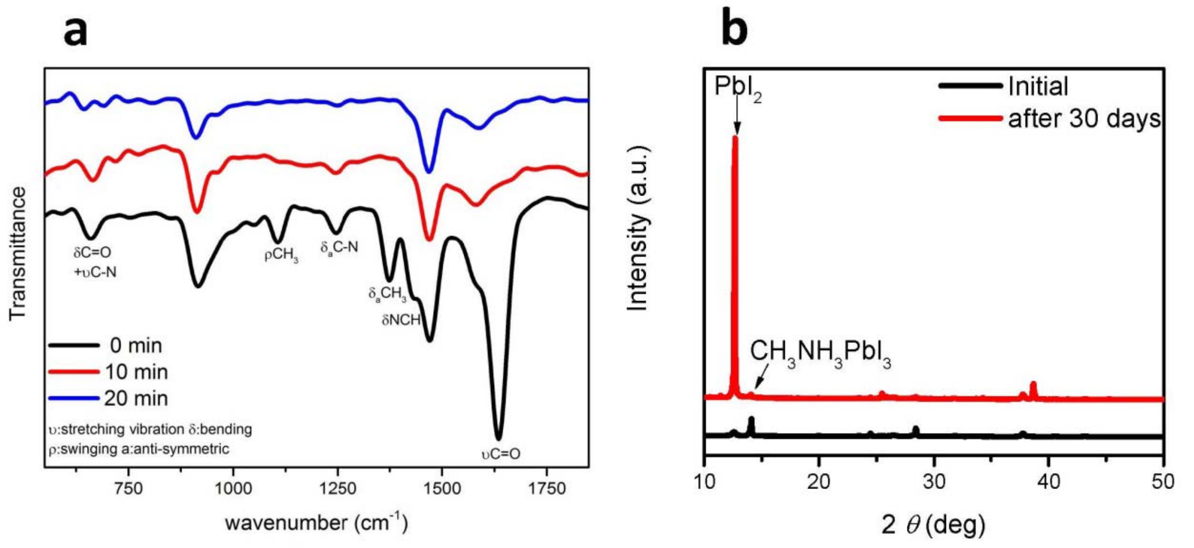

Figure S5 (a) FTIR spectrum of the two-step synthesized perovskite film with drying time of 0 min, 10 min and 20 min at 70 ^{o}C. The IR absorption peaks from DMF molecules are marked. (b) XRD patterns of the as-prepared perovskite film with drying time of 10 min at 70 ^{o}C (black line) and that after storing at ambient condition for 30 days (red line). It shows that with more residual DMF in the perovskite, the film decomposes more quickly.

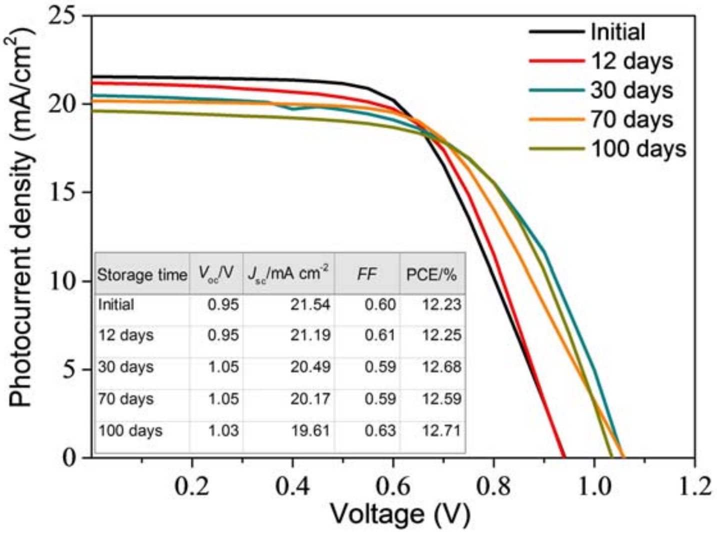

Figure S6 *J*-*V* curves and performance parameters of the solar cells fabricated with the aged perovksite film prepared from mCVT method.

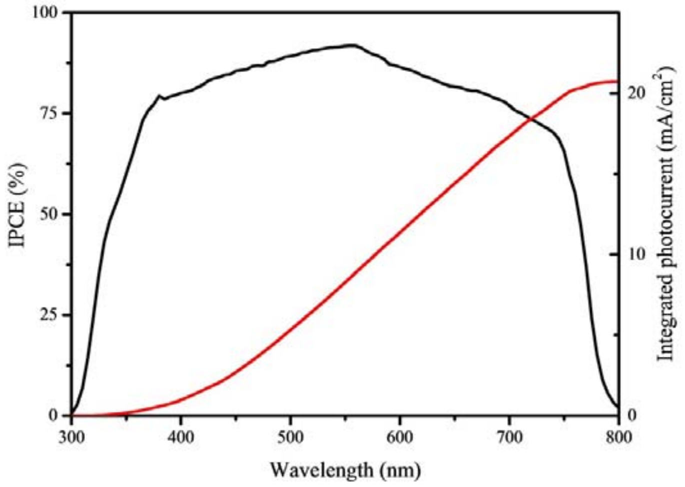

Figure S7 IPCE and integrated photocurrent of the as-fabricated perovskite solar cell from the mCVT $CH_3NH_3PbI_3$ film (the best device shown in Fig. 5 in main text).

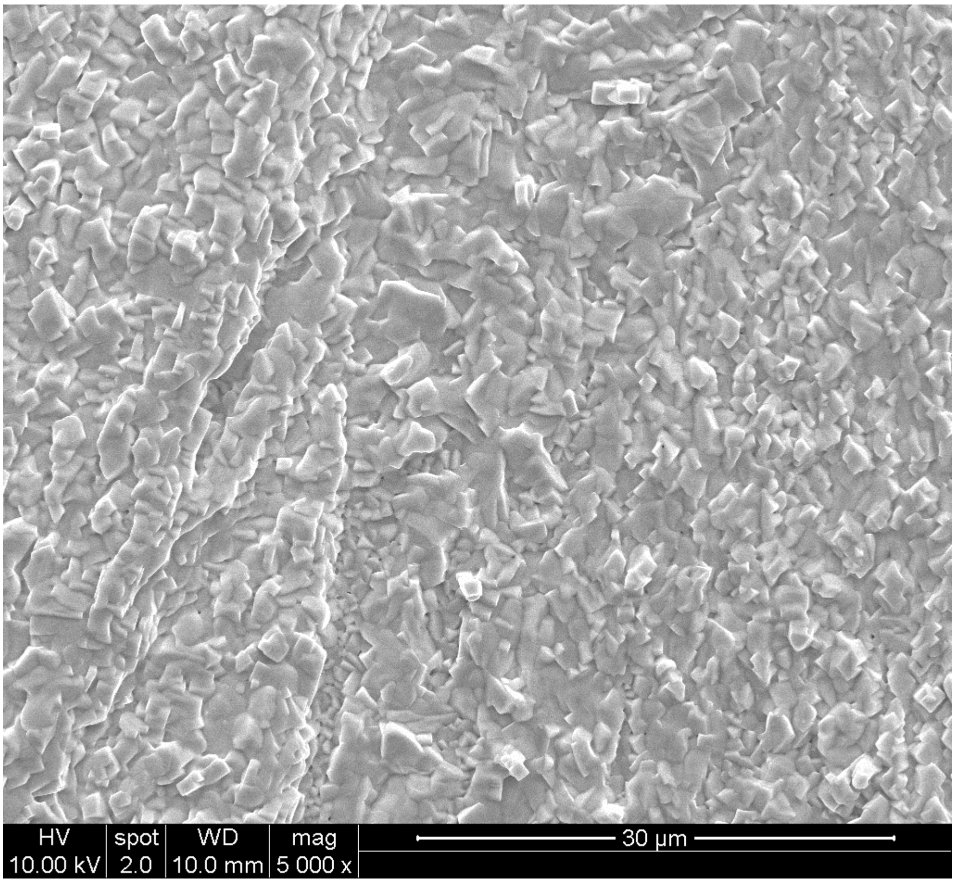

Figure S8 Large-area SEM characterization of the mCVT fabricated $CH_3NH_3SnI_3$ film, demonstrating the adaptability of the mCVT for the synthesis of high quality lead-free perovskite films.

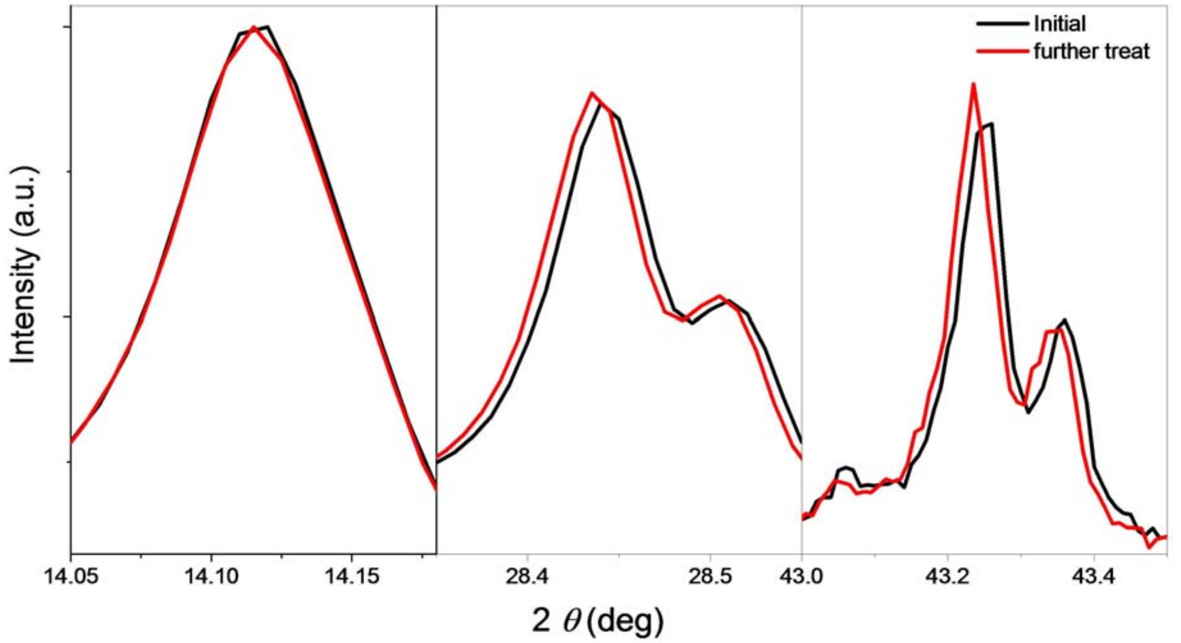

Figure S9 Selected XRD peaks of the one-step prepared perovskite film before (black line) and after further treatment through the mCVT reaction (red line).The diffraction peak position of (220), (314) and other planes are found to slightly left-shifted after the mCVT treatment, e.g. the diffraction peak of (220) plane changed from 28.44°to 28.43°. Based on the diffraction peak position, we can calculate that the lattice constant increased from 8.869 Å to 8.873 Å. This phenomenon is induced by the substitution of Cl in $CH_3NH_3PbI_{3-x}Cl_x$ with I, resulting in lattice expansion. If we assume that that the lattice constant changes linearly with the doping level of Cl, the doping level is estimated to be 0.1%. (5.675 Å for the lattice constant of $CH_3NH_3PbCl_3$).

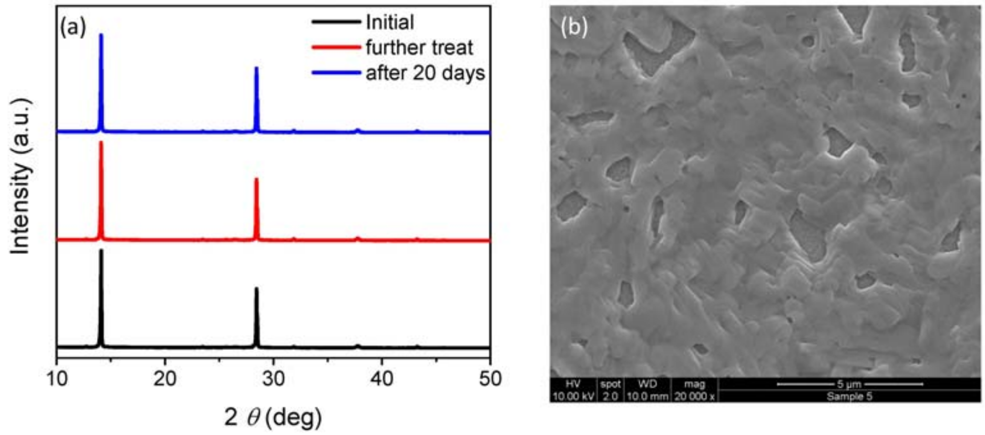

Figure S10 (a) XRD patterns of the perovskite film prepared from one-step method (black line), further treated with the mCVT method (red line) and storing at ambient condition for 20 days after the treatment (blue line) (b) SEM image of one-step-prepared perovskite film after further treated with the mCVT method.

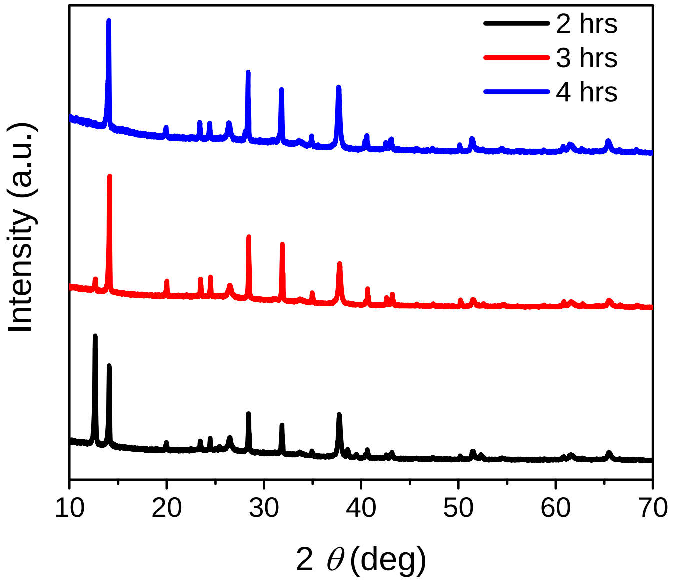

Figure S11 XRD patterns of the mCVT-prepared perovskite film using $PbCl_2$ and MAI as precursor at 140 °C for 2 h, 3 h and 4 h. The optimized reaction condition is 140 °C for 4 h.

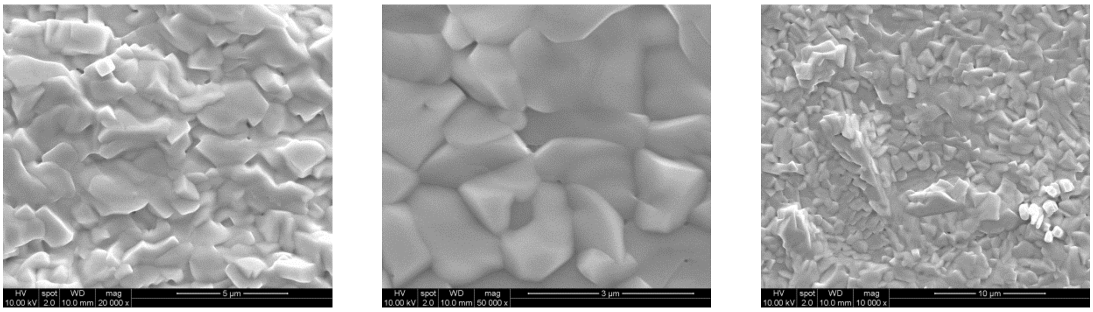

Figure S12 SEM images of the mCVT method prepared $CH_3NH_3SnI_3$ perovskite film using $SnCl_2$ and MAI as precursor.

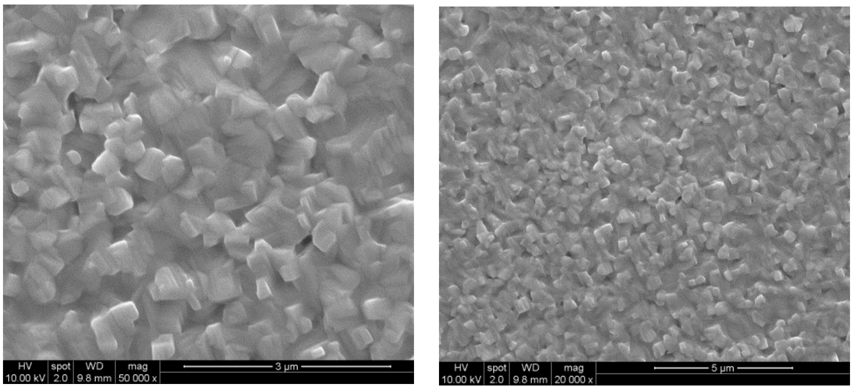

Figure S13 SEM images of the mCVT method prepared $CH_3NH_3PbBr_3$ perovskite film at 160 °C for 2 h using $PbBr_2$ and MABr as precursor.

6. References

[1] Kim, H. S., C. R. Lee, J. H. Im, K. B. Lee, T. Moehl, A. Marchioro, S. J. Moon, R. Humphry-Baker, J. H. Yum, J. E. Moser, M. Gratzel, N. G. Park, Sci Rep 2012, 2.
[2] Burschka, J., N. Pellet, S. J. Moon, R. Humphry-Baker, P. Gao, M. K. Nazeeruddin, M. Gratzel, Nature 2013, 499, 316.
[3] Lee, M. M., J. Teuscher, T. Miyasaka, T. N. Murakami, H. J. Snaith, Science 2012, 338, 643.
